# Factors affecting nutritional status among children aged below five years in Rwanda’s Western and Southern Provinces

**DOI:** 10.1186/s12889-024-19451-4

**Published:** 2024-07-30

**Authors:** Francois Xavier Sunday, Delice Niyigena Ilinde, Patrick Izabayo Rudatinya, Philemon Kwizera, Philbert Kanimba, Reverien Rutayisire, Maryse Umugwaneza

**Affiliations:** 1 School of Public Health, College of Medicine and Health Sciences, University of Rwanda, P O Box, Kigali, 3286 Rwanda; 2 School of Health Sciences, College of Medicine and Health Sciences, University of Rwanda, P O Box, Kigali, 3286 Rwanda

**Keywords:** Children under five years, Nutritional status, Stunting, Undernutrition, Underweight, Wasting

## Abstract

**Background:**

The state of a child’s nutrition is a critical indicator of their overall health and wellbeing. Public health still faces challenges from undernutrition, especially in developing nations across the globe. In Rwanda, around 33% of children aged under five years suffer from chronic undernutrition. Many factors, such as poverty, illiteracy, poor WASH practices, improper child feeding practices, and insufficient healthcare, are the leading causes of undernutrition. The study aims to assess infant and young child feeding practices, WASH, food security, and their association with the nutritional status of children under five years in Rwanda’s Western and Southern provinces.

**Methods:**

A community-based cross-sectional study design was applied to study factors affecting the nutritional status of children under five years in 439 households in the Karongi, Nyabihu, and Nyamagabe districts of Rwanda. The study assessed anemia, stunting, underweight, and wasting indicators, and collected data was analyzed using SPSS version 25.

**Results:**

The study findings indicate that among the children surveyed, 29.2% (128) were identified as stunted, 5.9% (26) were underweight, 2.3% (10) suffered from wasting, and 20.9% (31) had anemia. Factors associated with these conditions included larger household size [AOR = 2.108; 95% CI (1.016–4.371)], positively associated with stunting. Additionally, children from households where the head was above 60 years old were more likely to exhibit stunting [AOR = 4.809; 95% CI (1.513, 15.283)]. Furthermore, a high household dietary diversity score was positively linked to being underweight [AOR = 6.061; 95% CI (1.535,23.942)].

**Conclusion:**

Household characteristics like size, dietary diversity, and the age of the household head affect children’s nutritional status. Improving these conditions would enhance children’s nutritional status.

## Introduction

Undernutrition persists as a significant global health concern, particularly among children under five years. As of 2022, Child undernutrition remains alarmingly high on a worldwide scale, with 22.3% (148 million) children under age five experiencing stunting and 6.8% (45 million) facing wasting [1]. Africa has the highest stunting rate, hosting 31% (56 million) of stunted children [2]. Sub-Saharan Africa(SSA) partakes the highest burden of undernutrition compared to other sub-regions due to Poverty, food insecurity, political instability, climate variability, poor infrastructures, and inadequate feeding practices [3]. In Rwanda also undernutrition remains high, particularly with 33% of children under five years exhibiting stunting [4]. Consequently, malnourished children do not reach their full growth potential due to poor physical and cognitive development. Moreover, undernutrition has long-term severe health, social, and financial consequences. These children are more vulnerable to infectious and chronic illnesses because their immune systems are already compromised [5]. Addressing undernutrition in young children is essential to promote their health and well-being.

A multitude of factors affect the nutritional status of children under five years. They include global issues such as hunger affecting between 690 and 783 million and lack of increased and consistent access to nutritious food for approximately 2.4 billion individuals, predominantly residents of rural areas [6]. Moreover, half of undernutrition prevalence is attributed to poor WASH (Water, Sanitation, and Hygiene) practices due to the increased risk of diarrheal diseases, which hinder nutrient absorption [7]. Globally, 844 million people lack access to drinking water, and 2.3 billion do not have access to restrooms or other basic sanitation facilities [8]. During 2020, 80% of the households in Rwanda had access to an improved water source whereas only 35% of the households had an appropriate water treatment method. In addition, 72% of the households had access to improved toilet facilities [4].

Understanding the context-specific determinants of undernutrition is key to accelerating its reduction. However, knowledge gaps remain regarding how specific nutrition practices, food security, and WASH influence the nutritional status of children under five years old in Rwanda. This study aimed to fill this gap by investigating the impact of these factors on child nutrition in Rwanda’s Southern and Western regions. By exploring these relationships, the study sought to identify critical factors influencing child nutrition and provide evidence-based recommendations for policymakers, healthcare providers, and caregivers. The findings will contribute to the evidence base for designing effective interventions and policies to improve the nutritional status of young children in rural areas, ultimately fostering better health and developmental outcomes during early childhood.

## Methods

### Study design

This cross-sectional study was conducted in 2022 and employed a quantitative approach within Rwanda’s Western and Southern Provinces. Three districts were purposively chosen to host the study due to their notable prevalence of undernutrition, particularly among children under five years, as highlighted in the Rwanda Demographic and Health Survey (RDHS)2014–2015 [9]. The deliberate choice of these districts was grounded in the objective of capturing and analyzing the dynamics of undernutrition in regions identified as significant contributors to the broader challenge within the country.

### Study area

The study area encompassed three districts in Rwanda, each with unique geographical and agricultural features. Karongi district in the Western province, characterized by subsistence farming, has a population density of 380 people per square kilometer, with 83.2% of households engaged in agriculture [10]. The study focused on four of its thirteen sectors: Gishyita, Mubuga, Murundi, and Twumba. Nyabihu district, also in the Western province, is known for its Irish potato cultivation. It has a population density of 642 people per square kilometer and is prone to erosion due to its steep, fertile slopes [11, 12]. The study included four of its twelve sectors: Bigogwe, Jenda, Kintobo, and Mukamira. Nyamagabe district in the Southern province, a tea plantation cluster, has acidic soils and significant forest coverage, including parts of Nyungwe National Park. It has a population density of 441 people per square kilometer, and the study focused on the sectors of Kibirizi, Kitabi, Tare, and Uwinkingi [13, 14].

### Population, sample calculation, and sampling process

The total number of participating households was calculated using Taro Yamane’s (1973) formula n = N/1 + N(e)2 [15]. Where n stands for the expected sample, N was the entire population doing farming in Karongi, Nyabihu, and Nyamagabe, equivalent to 214,024 households [16], and e is the margin of error, which has been fixed to 0.05. To find the total sample, researchers added a 10% non-response rate. Among households in the study area that satisfied the inclusion criteria of being farmers and having a child between 0 and 59 months, 439 households were selected.

Following this, three districts were purposively selected based on their primary farming practices: Karongi (subsistence farming), Nyabihu (Irish potato farming), and Nyamagabe (tea plantations). In each district, four sectors were also purposively chosen, and subsequently, villages were randomly selected using Excel’s rand between functions. From each village, four households were identified systematically from lists provided by community health workers. The primary respondents were mothers or caretakers of children under five years old.

### Data collection

Trained enumerators conducted face-to-face interviews with caregivers to collect data on household socio-demographic and economic characteristics, food security, children’s feeding practices, caregiver demographics (age, education, employment), and water and sanitation facilities (drinking water source and treatment methods). Using the Kobo Collect smartphone app, enumerators gathered data. They also performed anthropometric measurements, including recumbent length for children aged 0–23 months using a portable length/height board, and height measurements for older children. Weight measurements were taken using a SECA electronic weighing scale. Blood samples were also collected from children to assess anemia status. All participants provided written informed consent, and interviews were conducted in Kinyarwanda the participant’s native language.

### Determination of anemia

Five University of Rwanda Biomedical Laboratory graduates collected blood samples from the participant’s ring finger on the non-active arm (usually the left) for an anemia test using a HemoCue Hb 201 + analyzer. A lancet was used to collect finger capillary blood in an aseptic manner [17]. A second drop of blood was drawn with a Capillary Transfer Tube for analysis. Anemia was hemoglobin levels < 11 g/dl [4].

### Data analysis

This process started with downloading the data set from Kobo collect to an Excel sheet. Data analysts checked the Excel data set for completeness and then imported it into SPSS version 25 for coding and analysis. The WHO Anthro software was used to calculate the z-scores for children’s length/height-for-age, length/height-for-weight, and weight-for-age. Children whose length/height-for-age z-score was below − 2 SD were classified as stunted, those with a length-for-weight z-score below − 2 SD were categorized as wasted, and those with a weight-for-age z-score below − 2 SD were considered as underweight [17]. Infant and Young Children’s Feeding practices were also assessed in this study [18], including early initiation of breastfeeding (EIBF), which is the proportion of children aged 0-23months who received breastmilk in the first hour of life, exclusive breastfeeding (EBF) that refers to the proportion of children aged from zero to five months who receive only breastmilk and introduction of solids, semi-solids or soft foods (ISSSF) which is the proportion of children who started complementary feeding between six and eight months. In addition, the percentage of children aged 12 to 23 months who received breastmilk in the previous 24 h represents continued breastfeeding (CBF). Minimum Meal Frequency (MMF) is the prevalence of children aged six to eight months who ate two times per day and three times for children aged 9 to 23 months among breastfed children and four times for non-breastfed children. Minimum Dietary Diversity (MDD) was the prevalence of children who consumed at least five food groups in the previous 24 h. Minimum Acceptable Diet (MAD) is the prevalence of children who received MMF and MDD during the last 24 h.

Participants’ handwashing practices were assessed by asking whether they washed their hands using clean water and soap at the five critical points [19]. Those who washed their hands during four to five critical times had good handwashing practices. In contrast, those who washed only at two to three critical times had fair hand washing, and the remaining group who washed their hands during one critical time had poor hand washing. Those who treated drinking water by boiling or other purification chemicals had adequate treatment methods. In contrast, those who used a clean cloth or didn’t use anything were classified as having inadequate water treatment methods [5]. Statisticians conducted descriptive statistics, providing frequencies and percentages. Bivariate analysis was performed using the chi-square or Fisher exact tests where necessary. Variables that exhibited significant association with a p-value ≤ 0.05 in the bivariate analysis were sent to a binary logistic regression model, thereby analyzing the nature of the association.

### Ethical considerations

The University of Rwanda Institutional Review Board granted ethical clearance for the study with ethics approval Notice: No 259/CMHS IRB 2021. The study provided individual informed consent after being told about the study’s design, goals, and significance. Participants were assigned codes to protect their privacy.

## Results

A total of 439 households took part in this survey; of them, 30.8% (135) were from Nyabihu, 35.1% (154) from Nyamagabe, and 34.2% (150) from Karongi with children under five years old and engaged in farming practices. There were 51.5% (226) boys among the children, while 48.5% (213) were girls. Also, 30.5% (134) of the children aged between six and 23 months, and only 7% (31) were below six months. Regarding the child caregivers, 45% (198) were between 25 and 34 years old. Moreover, 57.2% (252) of the caregivers had only attended primary education, whereas 11.6% (51) had no formal education. Additionally, 75.6% (332) of the child caregivers were engaged in farming and rearing livestock activities, whereas 15% (66) worked as casual laborers. Many of the households, 49.4% (217), were medium-sized, composed of four to seven members. Most of the study’s participants were located in the second and third wealth categories at 49.4% (217) and 42.1% (185), respectively. Most of the study participants, 74.5% (325), used adequate water treatment methods, whereas only 25.5% (112) applied inadequate water treatment methods. In addition to that, poor handwashing was practiced by 19.8% (87), 46.5% (204) practiced fair hand washing, and 33.7% (148) had good hand washing practices. (Table 1)

**Table 1:** Socio-demographic and economic characteristics of study participants

Characteristics of children	Frequency(*n*)	Percentage(%)
**Region**		
Karongi	150	34.2
Nyabihu	135	30.8
Nyamagabe	154	35.1
**Child sex**		
Girl	213	48.5
Boy	226	51.5
**Child age in months**		
0–5	31	7.0
6–23	134	30.5
24–35	117	26.7
36–47	99	22.6
48–60	58	13.2
**Mother’s age**		
17–24 years	52	11.8
25–34 years	198	45.1
35 and above	186	42.4
**Mother’s education**		
No formal education	51	11.6
Primary	251	57.2
Secondary and above	137	31.2
**Mother’s employment**		
Salary/pension	6	1.4
Petty trade and or handcrafts	9	2.1
Casual labor	66	15
Farming and Rising livestock	332	75.6
Housewives/unemployed	26	5.9
**Household size**		
Small size < 4	158	36
Medium size > 4 < 7	217	49.4
Large size > 7	64	14.6
**Wealth category**		
First wealth category	36	8.2
Second wealth category	217	49.4
Third wealth category	185	42.1
Fourth wealth category	1	0.2
**Drinking water treatment**		
inadequate treatment method	112	25.5
adequate water treatment	327	74.5
**Hand washing practice**		
Poor hand washing	87	19.8
Fair hand washing	204	46.5
Good hand washing	148	33.7

### Infant and young children feeding practices (IYCF) among children aged 0 to 23 months

Infants and young child feeding practices are presented in Table 2, which illustrates that most of the children, 80% (132), were breastfed within the first hour after birth (EIBF). Nyamagabe had the highest rate of EIBF at 84.9% (45), followed by Nyabihu district at 81.1% (43) and then Karongi at 74.6% (44). Among 31 children aged 0 to 5 months, 96.3% (30) received exclusive breastfeeding (EBF). Karongi and Nyabihu districts had an exclusive breastfeeding of 100% (9 and 13), while Nyamagabe districts had an exclusive breastfeeding rate of 90% (9). Among children aged six to eight months, caregivers introduced solids, semi-solids, and soft foods (ISSSF) to 73.7% (14). Karongi district had the highest ISSSF prevalence at 85.7% (6), followed by Nyamagabe with 83.3% (5), and Nyabihu had the least prevalence at 50% (3). Only 30.8% (41) of all children aged six to 23 months achieved a MDD of five out of eight food groups. By district, Nyamagabe had the least MDD at 9.3% (4), followed by Karongi with 34% (17) and Nyabihu district with 50% (20). Among all children aged six to 23 months, 63.9% (85) had MMF. Nyamagabe district had the highest MMF at 76.7% (33), followed by Nyabihu with 60% (24) and Karongi district with 56% (28). Almost one in five 18.8% (25) of all the children aged six to 23 months had only achieved a MAD. By district, Nyamagabe had the lowest MAD at 7% (3), followed by Karongi at 20% (10), and Nyabihu had the highest MAD level at 30% (12). A meager percentage of children aged from six to 23 months, about 12.8% (17), had consumed eggs and fresh foods (EFF) in the previous 24 h. Karongi district had the highest level of EFF of 20% (10), followed by Nyabihu district at 15% (6) and Nyamagabe with the least EFF at 2.3% (1). Most of the children, 91.8% (90) aged 12 to 23 months, were still breastfed (CBF). By district, Karongi district had the highest CBF rate at 97.2% (35), followed by Nyabihu district with 96.3% (26) and Nyamagabe district with 82.9% (29).

**Table 2:** Infant and young children feeding practices

Feeding practices		Karongi	Nyabihu	Nyamagabe	Total
		*n*(%)	*n*(%)	*n*(%)	*n*(%)
EIBF	No	15(25.4)	10(18.9)	8(15.1)	33(20)
	Yes	44(74.6)	43(81.1)	45(84.9)	132(80)
EBF	No	0	0	1(10)	1(3.1)
	Yes	9(100)	13(100)	9(90)	30(96.9)
ISSSF	No	1(14.3)	3(50)	1(16.7)	5(26.3)
	Yes	6(85.7)	3(50)	5(83.3)	14(73.7)
MDD	No	33(66)	20(50)	39(90.7)	92(69.2)
	Yes	17(34)	20(50)	4(9.3)	41(30.8)
MMF	No	22(44)	16(10)	10(23.3)	48(36.1)
	Yes	28(56)	24(60)	33(76.7)	85(63.9)
MAD	No	40(80)	28(70)	40(93)	108(81.2)
	Yes	10(20)	12(30)	3(7)	25(18.8)
EFF	No	40(80)	34(85)	42(97.7)	116(87.2)
	Yes	10(20)	6(15)	1(2.3)	17(12.8)
CBF	No	1(2.8)	1(3.7)	6(17.1)	8(8.1)
	Yes	35(97.2)	26(96.3)	29(82.9)	90(91.8)

*EIBF* Early Initiation of Breastfeeding, *EBF* Exclusive Breastfeeding, *ISSSF* Introduction of Solids and Semi-Solid Foods, *MDD* Minimum Dietary Diversity, *MMF* Minimum Meal Frequency, *MAD* Minimum Acceptable Diet, *CBF* Continued Breastfeeding

### Nutritional status

From the findings, 2.3% (10) of the children exhibited wasting, as Table 3 illustrates. Nyamagabe had the highest wasting rate of 2.6% (4), Karongi had the lowest wasting rate of 2% (3), and Nyabihu district had 2.2% (3). Underweight affected 5.9% (26) of the children. Karongi had the highest underweight rate of 8% (12), followed by Nyamagabe with 5.2% (8), and Nyabihu had the lowest underweight rate at 4.4% (6). A sum of 29.2% (128) of the children were stunted; among them, 31.3% (47) were from Karongi, 29.6% (40) were from Nyabihu, and 26.6% (41) of the stunted children were from Nyamagabe. Among 434 children who were tested for anemia, 12.9% (56) suffered from mild anemia, 7.8% (34) had moderate anemia, and 0.2% (1) had severe anemia. Karongi hosted the highest prevalence of mild and moderate anemic children at 20.9% (31) and 10.8% (16), respectively, followed by Nyamagabe with 13.2% (20) and 10.6% (16), respectively. Nyabihu district had 3.7% (5) mild anemic children and 1.5% (2) moderate anemic children. Only Nyamagabe district had one case of severe anemia.

**Table 3:** Child nutritional status

Nutritional status indicator		Karongi	Nyabihu	Nyamagabe	Total
		*n*(%)	*n*(%)	*n*(%)	*n*(%)
Wasting	Not wasted	147(98)	132(97.8)	150(97.4)	429(97.7)
	Wasted	3(2)	3(2.2)	4(2.6)	10(2.3)
Underweight	Normal	138(92)	129(95.6)	146(94.8)	413(94.1)
	Underweight	12(8)	6(4.4)	8(5.2)	26(5.9)
Stunting	Normal	103(68.7)	95(70.4)	113(73.4)	311(70.8)
	Stunted	47(31.3)	40(29.6)	41(26.6)	128(29.2)
Anemia status	No anemia	101(68.2)	128(94.8)	114(75.5)	343(79)
	Mild anemia	31(20.9)	5(3.7)	20(13.2)	56(12.9)
	Moderate anemia	16(10.8)	2(1.5)	16(10.6)	34(7.8)
	Severe anemia	0	0	1(0.7)	1(0.2)

### Socio-demographic characteristics and nutritional status

Socio-demographic characteristics and nutritional status cross-tabulations are presented in Table 4, illustrating that the caregiver’s and household head’s age were associated with a child being underweight with a p-value of 0.042 and 0.047, respectively. Household size was significantly associated with underweight and stunting, with p-values of 0.024 and 0.001, respectively. Household dietary diversity was found to be significantly associated with wasting and underweight, with p-values of 0.039 and 0.03, respectively.

**Table 4:** Association between socio-demographic characteristics and nutritional status

Variable		Wasting *n*(%)			Underweight *n*(%)			Stunting *n*(%)		
		No	Yes	*p*-value	No	Yes	*p*-value	No	Yes	*p*-value
Child age (month)	0 to 5	31 (96.9)	1 (3.1)	0.77*	31(96.9)	1(3.1)	0.092	25(78.1)	7 (21.9)	0.139
	6 to 23	133(97.8)	3 (2.2)		123(90.4)	13(9.6		88(64.7)	48(35.3)	
	24 to 60	265(97.8)	6(2.2)		259(95.6)	12(4.4)		198(731)	73 (26.9)	
Caregiver’s age (years)	17–24	52 (100)	0 (0)	0.435*	50 (96.2)	2 (3.8)	0.042	36 (69.2)	16 (30.8)	0.672
	25–34	194 (98)	4 (2)		180(90.9)	18 (9.1)		137(69.2)	61 (30.8)	
	≥ 35	180(96.8)	6 (3.2)		180(96.8)	6 (3.2)		136(73.1)	50 (26.9)	
Household’s head age	below3	68 (100)	0	0.442*	67 (98.5)	1 (1.4)	0.047	56 (82.3)	12 (17.6)	0.001
	30–39	182(97.8)	4 (2.1)		178(95.6)	8 (4.3)		140(75.2)	46 (24.7)	
	40–59	158(96.3)	6 (3.6)		150(91.4)	14 (8.5)		106(64.6)	58 (35.3)	
	60 and above	21 (100)	0 (0)		18 (85.7)	3 (14.2)		9 (42.8)	12 (57.1)	
Caregiver’s education	Higher education	4 (100)	0	0.341*	4 (100)	0	0.975*	3 (75)	1 (25)	0.53
	never attended	50 (98)	1 (1.9)		48 (94.1)	3 (5.8)		35 (68.6)	16 (31.3)	
	incomplete primary	140(96.5)	5 (3.4)		136(93.7)	9 (6.2)		98 (67.5)	47 (32.4)	
	incomplete secondary	79 (96.3)	3 (3.6)		78 (95.1)	4 (4.8)		63 (76.8)	19 (23.1)	
	complete primary	106 (100)	0		100 (94.3)	6 (5.6)		72 (67.9)	34 (32)	
	complete secondary	50 (98.)	1 (1.9)		47 (92.1)	4 (7.8)		40 (78.4)	11 (21.5)	
Household head education	none	49 (96.1)	2 (3.9)	0.334*	45 (88.2)	6 (11.8)	1.115	37 (72.5)	14 (27.5)	0.665
	primary	270(97.5)	7 (2.5)		261(94.2)	16 (5.8)		192(69.3)	85 (30.7)	
	secondary	110(91.1)	1 (0.9)		107(96.4)	4 (3.6)		82 (73.9)	29 (26.1)	
Household size	small	154 (97.5)	4 (2.5)	0.212	155 (98.1)	3 (1.9)	0.024	126 (79.7)	32 (20.3)	0.001
	medium	214(98.6)	3 (1.4)		200(92.2)	17 (7.8)		149(68.7)	68 (31.3)	
	large	61 (95.3)	3 (4.7)		58 (90.6)	6 (9.4)		36 (56.3)	28 (43.8)	
Household dietary diversity score	low	195(99.5)	1 (0.5)	0.039	188(95.9)	8 (4.1)	0.03	146(74.5)	50 (25.5)	0.32
	medium	213(96.4)	8 (3.6)		207(93.7)	14 (6.3)		150(67.9)	71 (32.1)	
	high	21 (95.5)	1 (4.5)		18 (81.8)	4 (18.2)		15 (68.2)	7 (31.8)	
Food security	Food secure	26 (92.9)	2 (7.1)	0.075	25 (89.3)	3 (10.7)	0.396	19 (67.9)	9 (32.1)	0.83
	Food insecure	403(98.1)	8 (1.9)		388(94.4)	23 (5.6)		292 (71)	119 (29)	
Hand washing	Poor	86 (98.9)	1 (1.1)	0.098*	83 (95.4)	4 (4.6)	0.806	64 (73.6)	23 (26.4)	0.673
	Fair	196(96.1)	8 (3.9)		192(94.1)	12 (5.9)		146(71.6)	58 (28.4)	
	Good	147(99.3)	1 (0.7)		138(93.2)	10 (6.8)		101(68.2)	47 (31.2)	
Drinking water treatment	Inadequate	110(98.2)	2 (1.8)	0.744	104(92.9)	8 (7.1)	0.643	76 (67.9)	36 (32.1)	0.47
	Adequate	319(97.6)	8 (2.4)		309(94.5)	18 (5.5)		235(71.9)	92 (28.1)	
Caregiver’s employment	Other	2 (100)	0 (0)	0.145*	2 (100)	0 (0)	0.545*	1 (50)	1 (50)	0.05*
	Unemployed	25 (96.2)	1 (3.8)		25 (96.2)	1 (3.8)		17 (65.4)	9 (34.6)	
	Casual labor	64 (97)	2 (3)		62 (93.9)	4 (6.1)		39 (59.1)	27 (40.9)	
	Farming	326(98.2)	6 (1.8)		312 (94)	20 (6)		242(72.9)	90 (27.1)	
	Arts	9 (100)	0 (0)		9 (100)	0 (0)		9 (100)	0 (0)	
	Salary/pension	3 (75)	1 (25)		3 (75)	1 (25)		3 (75)	1 (25)	
Wealth categories	First	35 (97.2)	1 (2.8)	0.622*	34 (94.4)	2 (5.6)	0.913*	30 (83.3)	6 (16.7)	0.092
	Second	211(97.2)	6 (2.8)		203(93.5)	14 (6.5)		148(68.2)	69 (31.8)	
	Third	182(98.4)	3 (1.6)		175(94.6)	10 (5.4)		133(71.9)	52 (28.1)	
	Fourth	1 (100)	0		1 (100)	0		0	1 (100)	

### Feeding practices and nutritional status

As observed in Table 5, there was no significant association between feeding practices and nutritional status with all P-values above 0.05.

**Table 5:** Association between feeding practices and nutritional status

		Wasting *n*(%)			Underweight *n*(%)			Stunting *n*(%)		
		No	Yes	*p*-value	No	Yes	*p*-value	No	Yes	*p*-value
EIBF	No	12(92.3)	1(7.7)	0.363*	11(84.6)	2(15.4)	0.591*	7(53.8)	6(46.2)	0.706
	Yes	51(98.1)	1(1.9)		48(92.3)	4(7.7)		31(59.6)	21(40.4)	
EBF	No	3(75)	1(25)	0.125*	3(75)	1(25)	0.125*	3(75)	1(25)	1*
	Yes	28(100)	0		28(100)	0		22(78.6)	6(21.4)	
MDD	No	92(97.9)	2(2.1)	1*	84(89.4)	10(10.6)	0.522	62(66)	32(34)	0.648
	Yes	41(97.6)	1(2.4)		39(92.9)	3(7.1)		26(61.9)	16(38.1)	
MMF	No	54(100)	0	0.276*	50(92.6)	4(7.4)	0.489	37(68.5)	17(31.5)	0.45
	Yes	79(96.3)	3(3.7)		73(89)	9(11)		51(62.2)	31(37.8)	
MAD	No	103(98.1)	2(1.9)	0.543	96(91.4)	9(8.6)	0.471	67(63.8)	38(36.2)	0.687
	Yes	30(96.8)	1(3.2)		27(87.1)	4(12.9)		21(67.7)	10(32.3)	

### Factors associated with children’s nutritional status

The variables significantly associated with nutritional status were sent to a binary logistic regression model to identify the nature of the association. Table 6 shows that the household’s head age was positively associated with underweight. Children living in households with their heads aged above 60 years were 11 times more likely to be underweight than their counterparts from households led by younger people. However, the association was not statistically significant. Moreover, household size was significantly associated with being underweight. Children from medium-sized households (4 to 7 members) were 4.3 times more likely to be underweight, and children from larger-sized households were 5.345 times more likely to be underweight. However, in the adjusted model, the association became statistically insignificant. Furthermore, household dietary diversity was also significantly associated with underweight. Children from households with a medium dietary diversity score were 1.5 times more likely to be underweight than those with low dietary diversity. Moreover, children from households with the highest dietary diversity were 5.2 times more likely to be underweight.

Household characteristics, including the age of the household head and household size, were found to have a significant positive association with stunting. In the adjusted model children from households headed by heads aged 30–39 years were 1.5 times more likely to be stunted than children from household heads below 30 years. Being in a household headed by someone aged 40–59 years increased the risk of stunting by 2.5 times. Living in households headed by persons aged 60 years and above increased the risk of stunting by 6.2 times. This association remained statistically significant in the adjusted model where children from households headed by people above 60 years were 4.8 times more likely to be stunted than those from households headed by younger people below 30 years. Moreover, children from large households were two times more likely to be stunted compared to their counterparts from smaller households.

Wasting was also found to be positively associated with Household dietary diversity. However, this association was not significant in the unadjusted model. Children from households with the highest dietary diversity were nine times more likely to be wasted than those from households with low dietary diversity. Besides, children from households with medium dietary diversity were 7.3 times more likely to be wasted.

**Table 6:** Binary logistic regression model

			Unadjusted model				Adjusted model			
Variable			*P* value	OR	95% C.I.		*P* value	OR	95% C.I	
					Lower	Upper			Lower	Upper
Underweight	Caregiver’s age	17–24		1			-	-	-	-
		25–34	0.229	2.500	0.561	11.138	-	-	-	-
		≥ 35	0.827	0.833	0.163	4.256	-	-	-	-
	Household’s head age	< 30		1						
		30–39	0.303	3.011	0.370	24.535	-	-	-	-
		40–59	0.080	6.253	0.806	48.531	-	-	-	-
		≥ 60	0.042	11.167	1.095	113.881	0.126	7.278	0.574	92.239
	Household size	≤4		1				1		
		> 4 < 7	0.020	4.392	1.264	15.255	0.112	3.005	0.775	11.652
		> 7	0.021	5.345	1.294	22.076	0.267	2.480	0.498	12.337
	Household dietary diversity	Low		1				1		
		Medium	0.308	1.589	0.652	3.874	-	-	-	-
		High	0.012	5.222	1.432	19.047	0.010	6.061	1.535	23.942
Stunting	Household’s head age	<30		1				1		
		30–39	0.236	1.533	0.756	3.109	-	-	-	-
		40–59	0.009	2.553	1.267	5.146	0.108	1.975	0.861	4.528
		60+	0.001	6.222	2.144	18.062	0.008	4.809	1.513	15.283
	Household size	≤4		1				1		
		> 4 < 7	0.017	1.797	1.109	2.911	0.191	1.441	0.833	2.495
		> 7	0.000	3.062	1.634	5.739	0.045	2.108	1.016	4.371
Wasting	Household dietary diversity	Low		1						
		Medium	0.062	7.324	0.908	59.090	-	-	-	-
		High	0.120	9.286	0.560	153.945	-	-	-	-

## Discussion

The study assessed the prevalence of malnutrition and factors influencing the nutritional status of children aged under five years in three districts of Rwanda, including Karongi and Nyabihu in the Western province and Nyamagabe in the Southern province. A high household dietary diversity score and household head’s age were significantly positively associated with underweight, whereas larger household size and household head’s age were positively associated with stunting. Despite the importance of Infant and Young Children Feeding Practices (IYCF), none of its indicators were found to be significantly associated with nutritional status.

Overall, 29.2% of the children were stunted, 5.9% were underweight, and 2.3% were wasted. The widespread presence of stunting was lower than 33% reported in the Rwanda Demographic and Health Survey (RDHS) 2019–2020. Underweight was also slightly below 8% in the RDHS, whereas wasting was higher than 1% reported in the RDHS [4]. The current study found 31.3% of the children in the Karongi district stunted, which is almost equal to 32% reported by RDHS. Karongi district had the highest stunting prevalence compared to Nyabihu and Nyamagabe districts, with 31.3%, 29.6%, and 26.6%, respectively. However, in the RDHS, Nyabihu district had the highest stunting rate at 46.7%, followed by Nyamagabe with 33.6% and Karongi with 32.4% [4]. This discrepancy might be associated with dietary intake, socioeconomic characteristics, and regional factors.

The current study reported low levels of anemia among children compared to the RDHS report. Moderate anemia affected 7.8% of the children, and 12.9% were mildly anemic, compared to 15% and 21% reported by the RDHS 2019–2020, respectively [4]. Karongi had the highest level of anemia compared to other regions, which may be attributed to the use of water from Lake Kivu in this district. The potential contamination or poor quality of this water source could significantly impact overall health [20]. For instance, a secondary study from 47 demographic and health surveys of different countries reported that children from households using unimproved sources of water or water sources were more likely to be anemic than their counterparts from households with improved water sources [21].

The current study reports no significant association between household food security status and any of the nutritional status indicators. This is contrary to the findings of a secondary study conducted in sub-Saharan Africa, which found a significant association between household food insecurity and stunting, where children from food-insecure households were more likely to be stunted than their counterparts [22]. Similarly, another study conducted in western Ethiopia also reported that children from food-insecure households were 2.25 times more likely to be underweight than their counterparts from food-secure households [23].

Household head’s age was positively associated with stunting among children. Children born from households headed by a person aged above 60 years were more likely to be stunted than their counterparts headed by younger individuals. This association may be attributed to the fact that older household heads might have retired or have limited earning capacity, contributing to lower income and resources within the household, which may result in insufficient access to nutritious food, healthcare, and other essential needs for children’s well-being. In addition to financial constraints, older people may have health issues that limit their time and ability to work or care for their families.

Household dietary diversity was also found to be significantly associated with wasting and underweight. Children from households with medium dietary diversity were seven times more likely to be stunted than those from low dietary diversity households’. Children from homes with high dietary diversity had six times higher odds of becoming underweight than those with low dietary diversity. This is contrary to another study conducted in Burkina Faso that reported no association between household dietary diversity and wasting [24]. Moreover, another study conducted in Ghana also reported no association between household dietary diversity and stunting or wasting [25]. However, in Burkina Faso, household dietary diversity was negatively associated with stunting and underweight, whereas children from households with the highest dietary diversity were less likely to be stunted or underweight than their counterparts [24].

This study has shown that larger household size is a risk factor contributing to stunting, which is in agreement with other studies conducted in Sub-Saharan Africa, Nigeria, and Ethiopia [26–28]. This association can be attributed to increased food competition within larger households, making it more challenging to meet each household member’s individual nutritional needs. As a result, individuals’ nutritional needs, particularly those of vulnerable groups like children, may not be adequately met, leading to higher rates of undernutrition.

### Limitations

Recall bias was probably present since the data was collected based on the caregivers’ replies. In addition to that, anthropometric measurement bias might have arisen in the study; however, they were minimized by training and supervising data collectors. Although the study utilized a cross-sectional study design, it still offers valuable insights into the factors associated with children’s nutritional status.

## Conclusion

In conclusion, apart from wasting, the widespread presence of other nutritional status indicators, namely underweight and stunting, were below the prevalence reported by the RDHS 2019–2020. Infant and young children’s feeding practices, WASH practices, and household food security were not significantly associated with the child’s nutritional status. However, Household characteristics such as household size, household head’s age, and household dietary diversity were significantly associated with children’s nutritional status.

## Data Availability

The data used for writing this paper is available to whoever needs it for research purposes, it can be accessed by direct contact to authors for promoting transparency, collaboration and advancement of knowledge.
